# Aberrant Community Architecture and Attenuated Persistence of Uropathogenic *Escherichia coli* in the Absence of Individual IHF Subunits

**DOI:** 10.1371/journal.pone.0048349

**Published:** 2012-10-25

**Authors:** Sheryl S. Justice, Birong Li, Jennifer S. Downey, Shareef M. Dabdoub, M. Elizabeth Brockson, G. Duane Probst, William C. Ray, Steven D. Goodman

**Affiliations:** 1 Center for Microbial Pathogenesis, The Research Institute at Nationwide Children's Hospital, Columbus, Ohio, United States of America; 2 Department of Pediatrics and Urology, The Ohio State University College of Medicine, Columbus, Ohio, United States of America; 3 Division of Biomedical Sciences, Herman Ostrow School of Dentistry, University of Southern California, Los Angeles, California, United States of America; 4 Battelle Center for Mathematical Medicine, The Research Institute at Nationwide Children's Hospital, Columbus, Ohio, United States of America; Arizona State University, United States of America

## Abstract

Uropathogenic *Escherichia coli* (UPEC) utilizes a complex community-based developmental pathway for growth within superficial epithelial cells of the bladder during cystitis. Extracellular DNA (eDNA) is a common matrix component of organized bacterial communities. Integration host factor (IHF) is a heterodimeric protein that binds to double-stranded DNA and produces a hairpin bend. IHF-dependent DNA architectural changes act both intrabacterially and extrabacterially to regulate gene expression and community stability, respectively. We demonstrate that both IHF subunits are required for efficient colonization of the bladder, but are dispensable for early colonization of the kidney. The community architecture of the intracellular bacterial communities (IBCs) is quantitatively different in the absence of either IhfA or IhfB in the murine model for human urinary tract infection (UTI). Restoration of Type 1 pili by ectopic production does not restore colonization in the absence of IhfA, but partially compensates in the absence of IhfB. Furthermore, we describe a binding site for IHF that is upstream of the operon that encodes for the P-pilus. Taken together, these data suggest that both IHF and its constituent subunits (independent of the heterodimer), are able to participate in multiple aspects of the UPEC pathogenic lifestyle, and may have utility as a target for treatment of bacterial cystitis.

## Introduction

Many bacterial species multiply within organized communities as a part or whole of their lifestyles in the environment or in the host. Uropathogenic *Escherichia coli* (UPEC), the causative agent of up to 80% of all urinary tract infections (UTIs) [1], is one such species. UPEC uses a community-based developmental pathway to propagate within the cytoplasm of urothelial cells during bacterial cystitis [2]–[4]. The developmental pathway begins with attachment-mediated invasion into the superficial bladder epithelial cells via FimH binding to the mannosylated uroplakin proteins (Zhou et al., 2001) and involves fusiform vesicles [5], cyclic AMP [5], Toll-like receptor-4 (TLR4) [6] and integrins [7]. Within the cytoplasm, bacillary-shaped UPEC multiply within loosely associated intracellular bacterial communities (IBCs) [2]. IBC maturation involves both changes in cell division fidelity and community architecture, which culminates in an organized, globular community architecture composed primarily of UPEC in a coccoid-like morphology. Once the IBC occupies the majority of the cytoplasm, UPEC regain a bacillary shape, become motile and egress from the epithelial cell through disruptions in the cell membrane. The intracellular amplification of UPEC occurs in repetitive cycles through attachment of egressed organisms to naïve superficial epithelial cells and ultimately culminates in the establishment of a latent or chronic infection [8]–[10]. Evidence for each of these stages is observed in urine samples and bladder biopsies of patients colonized with either UPEC or *Klebsiella pneumoniae* [11], [12], which demonstrates that these events comprise the pathogenic lifestyle of multiple uropathogens during cystitis.

Integration Host Factor (IHF), a member of the ubiquitous DNABII family, acts as a DNA architectural element that can alter the conformation of nucleoprotein interactions inside the cell influencing replication, transcription, recombination and DNA repair [13]–[15]. IHF is required for regulation of a number of genetic loci associated with virulence. Production of elastase in *Vibrio vulnificus* [16], cholera toxin in *Vibrio cholera* [17], and type III secretion effectors in *E. coli* [18] require IHF. IHF promotes persistence of *Legionella pneumophila* in the protist host *Acanthamoeba castellanii* [19]. Regulation of adhesins and capsule is also IHF-dependent in *Neisseria* [20] and *E. coli* [21], [22].

The DNABII family of proteins is also observed in the extrabacterial milieu [23]–[25]. The extrabacterial accumulation of the *Streptococcus* DNABII family member, HlpA, elicits a proinflammatory immune response in macrophages [26] that may contribute to tissue damage associated with infection.

Extrabacterial DNA (eDNA) is a key component of communities formed by many pathogenic bacterial species [27]. The DNABII family is also critical for the integrity of bacterial communities that utilize eDNA as a structural component of the matrix. We demonstrated that antibodies directed against *E. coli* DNABII family members disrupt communities formed by multiple human pathogens under laboratory conditions [28]. Sequestration of extrabacterial DNABII family members from the community matrix also increased bacterial sensitivity to antimicrobials [28]. In addition, vaccination against IHF reduced the duration of established otitis media mediated by non-typeable *Haemophilus influenzae* in a mammalian model of human disease [28]. Therefore, extrabacterial DNABII members, appear to be a plausible target for prevention and/or treatment of community-based infectious diseases.

In this study, we investigate the contribution of one DNABII family member, IHF, to the pathogenic lifestyle of UPEC. Our studies unveil unique phenotypes associated with the presence of individual IHF subunits with regard to the regulation of type 1 pilus, as well as the overall architecture of the intracellular communities and colonization of the urinary tract. In addition, we identify an additional intrabacterial role for IHF in the production of the P-pilus. These studies demonstrate that IHF is essential for UPEC-mediated UTI and establishes functionality for each individual subunit of IHF in the absence of the canonical IHF heterodimer.

## Results

### Inactivation of *ihfA* and *ihfB* in UPEC

With our recent observation that IHF (referred to here as IhfAB) functions in the community matrix of a number of organisms including UPEC [28], we were interested to determine the role(s) of IhfAB during bacterial cystitis. IhfAB consists of 2 homologous subunits that form a heterodimer. In contrast to being able to make *null* alleles in genes encoding both subunits of IhfAB in the laboratory *E. coli* strain MG1655, we were unable to stably inactivate both subunits in our prototypical UPEC strain, UTI89 (Table 1). There were no significant differences in the growth rates of the remainder of the strains used in this study (data not shown). Mutations in either IHF subunit, *ihfA* or *ihfB*, have historically yielded a phenotype equivalent to that of the double mutant in laboratory adapted strains. Hence, the heterodimer has been presumed to be the only active form [29], [30]. However, individual subunits readily form functional homodimers in complex with DNA *in vitro* [29], [30], suggesting that homodimers may form upon stoichiometric changes in levels of the individual subunits. The inability to readily inactivate both genes in our clinical isolate suggests that the presence of one subunit may retain some functions that are essential for viability.

**Table 1:** Strains.

Strain Name	Genotype	Source
BW25113	*rrnB3 ÄlacZ4787 hsdR514 Ä*(*araBAD*)*567 Ä*(*rhaBAD*)*568 rph-1*	[71]
JW1702-1	BW25113 *ÄihfA786*::kan	[71]
JW0895-3	BW25113 *ÄihfB735*::kan	[71]
K1141	*ihfA11*::*Tn*10	*E. coli* Stock Center
MG1655	F^-^ lambda^-^ *ilvG*- *rfb*-50 *rph*-1	[72]
N99	*ÄgaIK*	[73]
SG83	N99 *ihfB*::Cam	[74]
SG84	N99 *ihfAÄ82* Tn10	[75]
SJ1000	UTI89 *surA*::kan	[76]
UTI89 *ΔkpsF*	Polar inactivation of region I	[44]
ROL607	MG1665 *ihfB*::Cam *ihfA11*::*Tn*10	This study
UTI89	Cystitis Clinical Isolate	[8]
	UTI89/pANT4	[2]
	UTI89/pMMB66	This study
ROL745	UTI89 *ihfA11*::*Tn*10	This study
	UTI89 *ihfA11*::*Tn*10/pHNá	This study
	UTI89 *ihfA11*::*Tn*10/pHNâ	This study
	UTI89 *ihfA11*::*Tn*10/pMMB66	This study
ROL747	UTI89 *ÄihfA786*::kan	This study
	UTI89 *ÄihfA786*:::kan/pHNá	This study
	UTI89 *ÄihfA786*:::kan/pHNâ	This study
ROL603	UTI89 *ihfB*::Cam	This study
	UTI89 *ihfB*::Cam/pHNá	This study
	UTI89 *ihfB*::Cam/pHNâ	This study
ROL748	UTI89 *ÄihfB735*::kan	This study
	UTI89 *ÄihfB735*::kan/pHNá	This study
	UTI89 *ÄihfB735*::kan/pHNâ	This study
	UTI89 *ÄihfB735*::kan/pMMB66	This study

The genotype of the strains and sources are indicated in the table.

### Type 1 piliation in the absence of IhfA or IhfB

**Figure 1:**
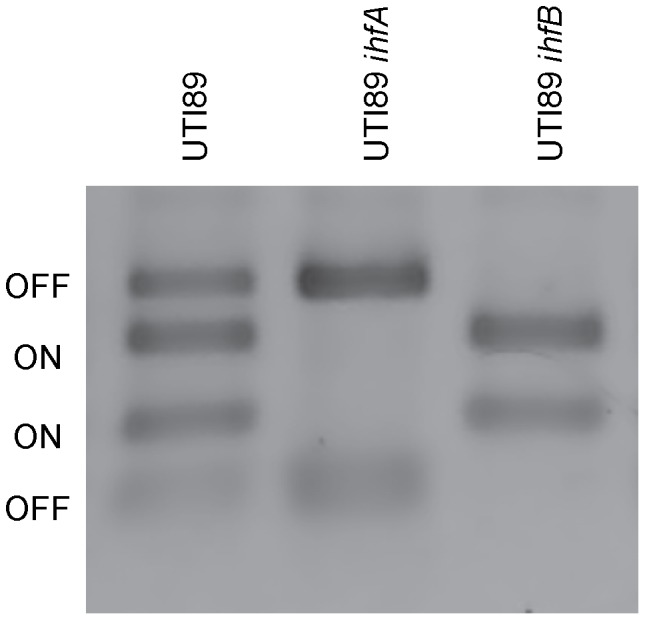
Promoter orientation. The promoter region (*fimS*) from UTI89, ROL745 (UTI89 *ihfA*) and ROL603 (UTI89 *ihfB*) were amplified by PCR and digested as previously described [65]. The size of the fragments following digestion indicates the orientation of the promoter (“off”  = 539 & 187 bp, while “on”  = 433 & 293 bp). A representative gel is shown to demonstrate the different orientations observed in the UTI89 wild type and mutant strains. The mutated genes are indicated above each lane.

Previous studies have demonstrated that the IhfA and IhfB heterodimer (IhfAB) participates in recombination events associated with promoter orientation of the *fim* operon (encoding type 1 pilus) in laboratory strains [22]. The type 1 pilus is essential for both bladder colonization and internalization [31]–[40]. Therefore, we wanted to carefully examine type 1 piliation phenotype of either *ihfA* or *ihfB* inactivation in our prototypical cystitis UPEC strain, UTI89. We examined each of the *ihf* mutations currently available (Table 2) to validate that the observed phenotypes were not allele specific. Type 1 piliation in UPEC strains defective in one or both subunits of IHF was determined using *in vitro* yeast agglutination assays. The ability of the bacteria to cross-link the yeast cells resulting in yeast cell aggregation is mediated by the type 1 pilus binding to the mannosylated proteins on the yeast surface. Type 1-pilus dependent yeast agglutination was not observed in any of the *ihfB* mutants (Table 2). Consistent with this observation, the promoter remains in the “off” state in the absence of IhfB (Table 2, Figure 1). Yeast agglutination was restored by the addition of *ihfB* (pHNβ) or *ihfAB* (pHNβα) *in trans* (Table 2). The overproduction of IhfA (pHNα) did not restore agglutination to the *ihfB* mutant, suggesting that increased protein concentration of the other homologous subunit is not sufficient to compensate for the absence of IhfB. Mannose sensitive yeast agglutination was diminished or ablated in the absence of IhfA (*ÄihfA786*::kan, *ihfA11*::*Tn*10; Table 2). As observed above, yeast agglutination was restored by the addition of *ihfA* (pHNα) or *ihfAB* (pHNβα) *in trans* (Table 2), indicating that the deficiency in piliation can be attributed to the absence of IhfA. In contrast to the strains deficient in IhfB, the promoter was present in the “on” position in the presence of either *ihfA* allele (Table 2, Figure 1), suggesting that IhfB may retain sufficient activity to initiate promoter conversion to the “on” orientation but is unable to support full piliation.

**Table 2:** Type 1 pilus phenotypes.

Genotype	Piliation	Promoter Orientation
MG1655	++	mix (off)
MG1665 *ihfB*::Cam *ihfA11*::*Tn*10	−	not done
UTI89	++	mix (even)
UTI89/pANT4	++	mix (even)
UTI89/pMMB66	+++	N.D.
UTI89 *ihfA11*::*Tn*10	−	off
UTI89 *ihfA11*::*Tn*10/pHNα	++	on
UTI89 *ihfA11*::*Tn*10/pHNβ	−	off
UTI89 *ihfA11*::*Tn*10/pMMB66	+++	N.D.
UTI89 *ΔihfA786*::kan	+	mix (off)
UTI89 *ΔihfA786*:::kan/pHNα	+++	on/off
UTI89 *ΔihfA786*:::kan/pHNβ	+	mix (off)
UTI89 *ihfB*::Cam	−	on
UTI89 *ihfB*::Cam/pHNα	−	on
UTI89 *ihfB*::Cam/pHNβ	+++	mix (even)
UTI89 *ΔihfB735*::kan	−	on
UTI89 *ΔihfB735*::kan/pHNα	−	on
UTI89 *ΔihfB735*::kan/pHNβ	+++	mix (even)
UTI89 *ΔihfB735*::kan/pMMB66	+++	N.D.

The relative level of type 1 piliation is indicated by the number of “+” signs. The orientation of the *fimS* promoter sequence is indicated. In cases where the promoter orientation was mixed in the culture, the predominant orientation is indicated in parentheses. N.D., not determined.

### Both IhfA and IhfB are required for colonization of the urinary bladder

**Figure 2:**
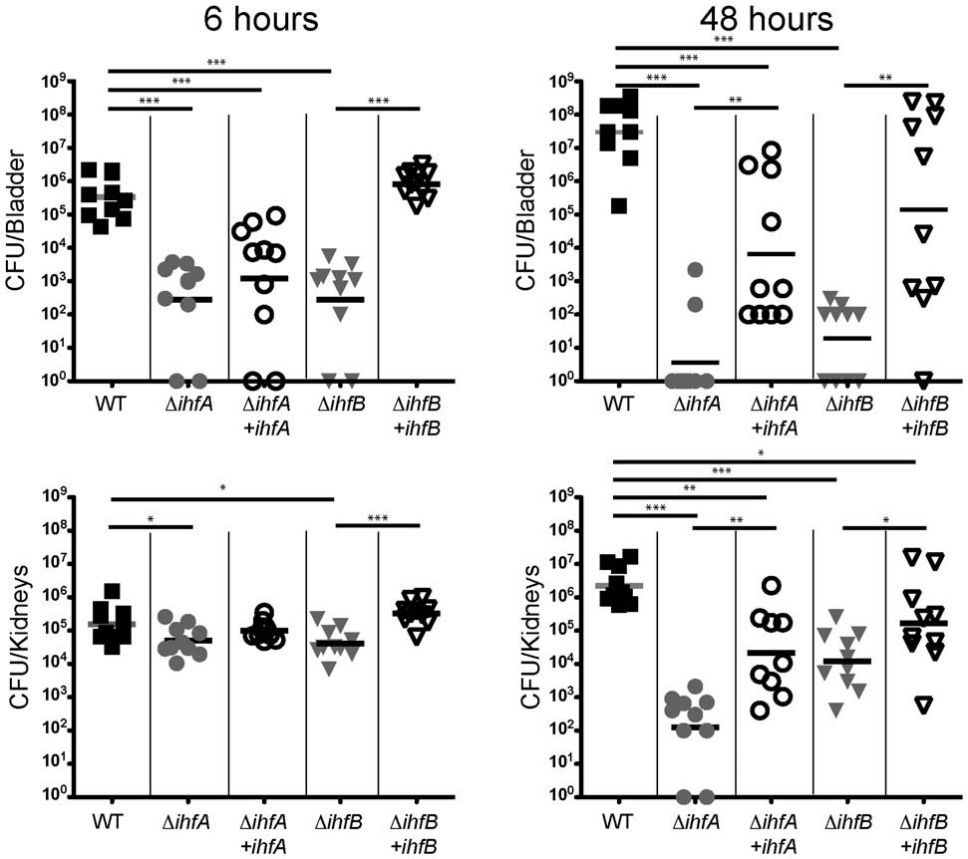
IhfAB subunits are required for UPEC pathogenesis. Each symbol represents single infected murine bladder or combined kidney pair. Female mice were infected with wild type UTI89 (black filled squares), ROL745 (UTI89 *ihfA11*; gray filled circles), ROL745/pHNα (UTI89 *ihfA11* complemented with *ihfA;* black open circles), ROL603 (UTI89 *ΔihfB;* gray filled triangles), or ROL603/pHNβ (UTI89 *ihfB::Cam* complemented with *ihfB;* black open triangles) for 6 or 48 hours post inoculation. Bacterial burden of bladders and kidneys was enumerated as colony forming units ( CFUs). Statistical significance determined using non-parametric Mann Whitney (*<0.04, ** = 0.018, *** = 0.0017, ****<0.0006).

We have previously demonstrated that extrabacterial IhfAB (eIhfAB) contributes to the stability of UPEC communities grown on glass surfaces *in vitro* [28]. We next investigated the contribution of the individual subunits to the intracellular community development observed during cystitis [2]. Female mice were transurethrally inoculated with UTI89, UTI89 *ihfA11*::*Tn*10 (ROL745), UTI89 Δ*ihfB* (ROL603) or the complemented mutant strains to determine the ability of each strain to persist in the urinary tract. Inactivation of either Ihf subunit resulted in a 3 log decrease in colonization of the bladder as early as 6 hours post infection (Figure 2, p = 0.0017). The attenuation of colonization was more severe in the bladder at 48 hours post infection, as evidenced by at least a 5 order of magnitude decrease in bacterial burden (p<0.0006) in both the *ihfA* and *ihfB* mutants compared to wild type UTI89 (Figure 2). There was a statistically significant increase in the persistence of UPEC when either IhfA or IhfB were produced *in trans* in the strains devoid of chromosomal *ihfA* or *ihfB*, respectively (Figure 2). There was a bimodal distribution of bacterial burden, approximately 45% of the bladders infected with either mutant strain exhibited UPEC colonization that was indistinguishable from that of the parental strain. In the bladders where the bacterial burden was not fully restored, the level was still one or two orders of magnitude greater than in the absence of complementation (Figure 2), indicating that IhfAB participates in functions that are critical for persistence in the host.

### Community architecture in the absence of IhfAB during cystitis

**Figure 3:**
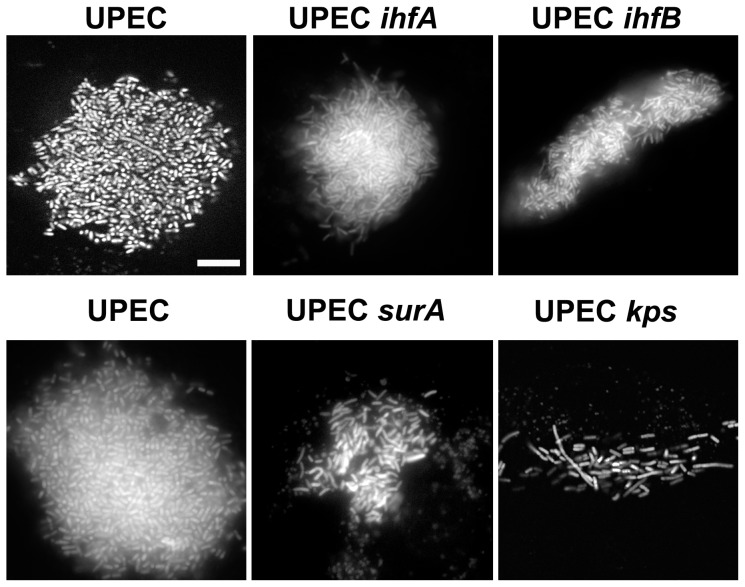
Architecture of Intracellular Bacteria. Female mice were infected with UTI89/pANT4, ROL745/pANT4 (UTI89 *ihfA11*), ROL603/pANT4 (UTI89 *ΔihfB*), SJ1000/pANT4 (UTI89 *surA*), or UTI89 *Δkps*/pANT4 for 6 hours. Bladders were prepared for visualization by fluorescent microscopy [41]. The intracellular characteristics of each of the strains indicated were visualized using strains that constitutively produce green fluorescent protein. Images were taken as an optical section (upper panel UPEC) or as total fluorescence of entire community (all other panels). The strains are indicated above each panel. Scale bar  = 10 µm and scale is unchanged between images.

**Figure 4:**
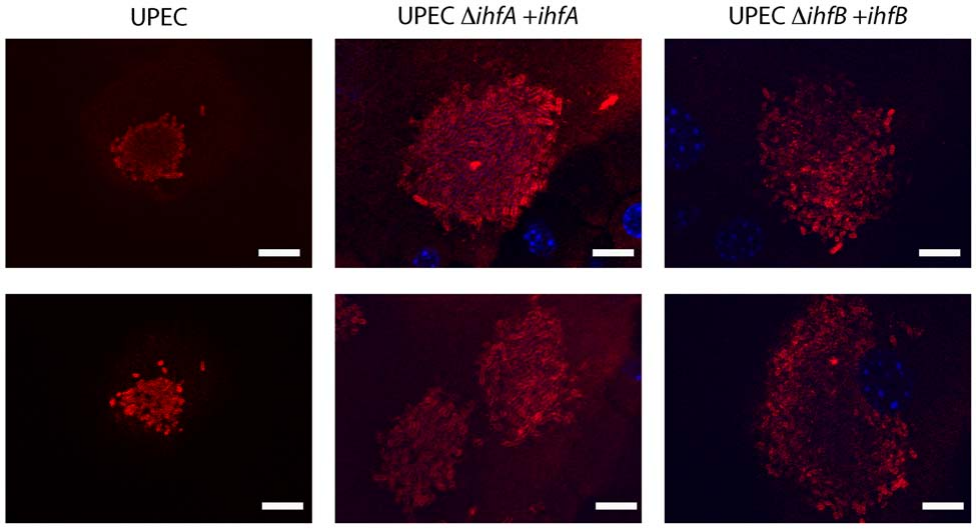
Community architecture is restored upon complementation in trans. Female mice were infected with UTI89, ROL745/ pHNá (UTI89 *ihfA11*), or ROL603/ pHNâ (UTI89 *ΔihfB*) for 6 hours. Bladders were prepared for visualization by immune-fluorescent microscopy [41]. Images were taken as an optical section. The strains are indicated above each panel. Scale bar  = 10 µm and scale is unchanged between images.

Previous microscopic evaluation of UPEC community development revealed important populations that are essential for persistence [2], [41], [42] in the bladder. Furthermore, the use of UPEC strains defective in specific bacterial traits results in variations in community development [43]. However, the regulatory mechanisms that control UPEC development are not understood. To gain more insight into the potential mechanisms that underlie the persistence defect by exploitation of the pleiotropic phenotypes associated with loss of IhfA or IhfB, bladders of mice infected with *ihfA* or *IhfB* deficient strains were analyzed by fluorescence microscopy. Introduction of the pANT4 plasmid for constitutive production of green fluorescent protein had no effect on the production of Type 1 pili or the orientation of the promoter (data not shown). Communities were evaluated at 6 hours post infection, when the transition from the rod to coccoid shape generally occurs [2], [33], [41]. In contrast to the potential for 100 s of communities formed with the parental strain, and in accordance with the observed bacterial burden, in the absence of either IhfA or IhfB only a few epithelial cells (≤2) contained communities but unlike the parent strain, their architecture was aberrant (Figure 3). Consistent with the increase in the bacterial burden (Figure 2), robust IBC development was fully restored by complementation of the gene of interest in *trans* (Figure 4). The lack of visible clusters of bacteria within the epithelial cells indicates that the growth of an organized community is severely crippled in the absence of either subunit. The observation that the developmental cycle of intracellular communities was restored in the presence of the gene *in trans* suggests that the defects in community development are due to the absence of the subunit in question. The defect in community development in the absence of proteins with pleiotropic phenotypes has been observed with other UPEC mutants that display normal growth rates under laboratory conditions [33], [39], [40], [44], [45].

**Figure 5:**
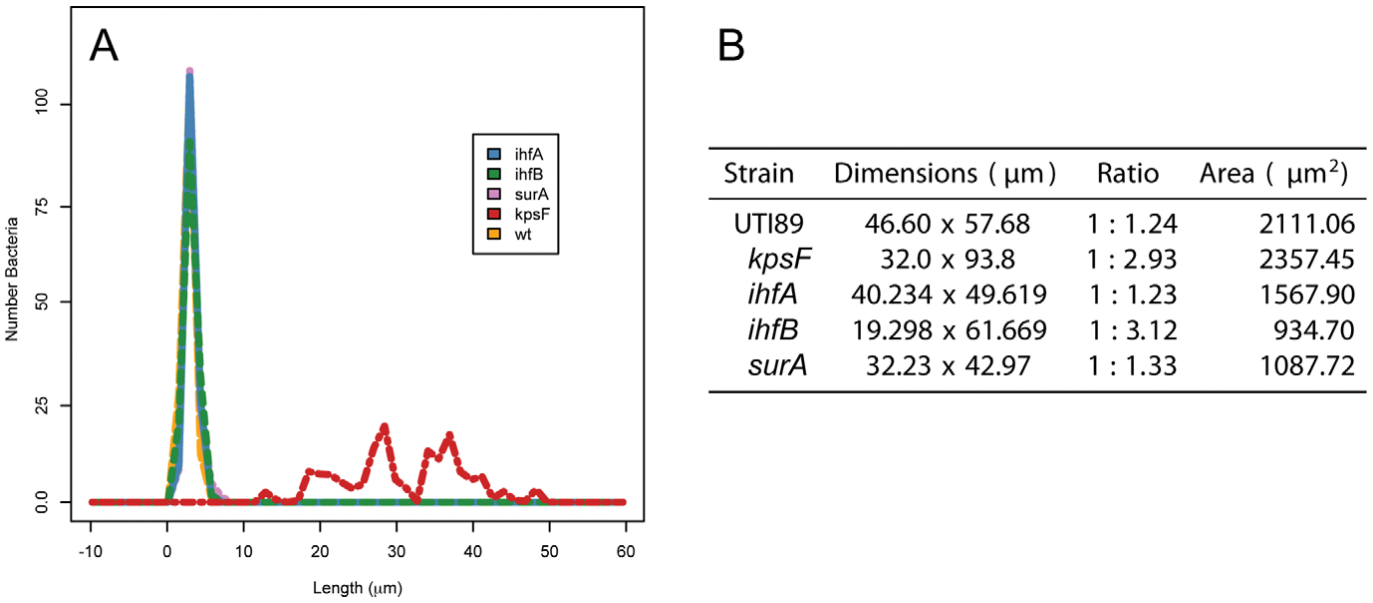
Quantitative Assessment of Intracellular Bacteria. The individual statistics of the bacteria growing within superficial mouse bladder epithelial cells as depicted in Figure 3 were determined. (A) The cell length of each bacterium was evaluated within individual images (NIH Image J) or 3-dimensional rendering [60]. The cell length distribution for each strain is represented. (B) The maximum dimension in the x and y axes, the ratio of the axes as well as the overall area of the intracellular bacteria were determined using Prokarymetrics.

In the few cases where intracellular clusters were observed, there were measurable visual differences as compared with prototypical IBCs (Figure 3). Although the bacterial burden of the UTI89 *ihfA11*::*Tn*10 and UTI89 *ΔihfB* strains was similar (Figure 2), the overall organization of the intracellular clusters is unique for each strain (Figures 3, 5). In the absence of capsule, the intracellular bacterial clusters have distinctive changes in both community architecture as well as bacterial morphology (Figure3, 5) [44]. In contrast to UPEC *Δkps*, there was no significant difference in the morphology of the individual bacteria deficient in either IHF subunit (Figure 5A), suggesting that the phenotypic changes associated with intracellular clusters of UTI89 *ihfA11*::*Tn*10 or UTI89 *ΔihfB* are not related to a defect in capsule synthesis. UTI89 *ihfA11*::*Tn*10 retains the ability to form the more globular-shaped community indicative of the middle stage community [2] as observed with UTI89 and UPEC *ΔsurA* (ratio of width to height ∼1.2) (Figure 5B). However, there was a decrease in the area occupied by the bacterial cluster, suggesting that robust intracellular growth may require IhfA. Moreover, the conversion to the more coccoid-shaped individuals was not observed, suggesting that targets of IhfAB contribute to the morphological changes associated with intracellular community maturation. In contrast, the shape and density of the community formed by UTI89 *ΔihfB* was more open and elongated, resembling that of UPEC *Δkps* (ratio of width to height ∼3) (Figure 5B). The increase in interbacterial distance and shape of the cluster suggests that IhfB is important in coordination of the community matrix or production of a surface structure that mediates bacterial interaction with the matrix. The differences in the architecture of the intracellular clusters formed in the absence of either subunit further suggests that the individual subunits retain functionality and that, although homologous, retain unique functions.

### Type 1 piliation does not restore virulence in the absence of IhfA

**Figure 6:**
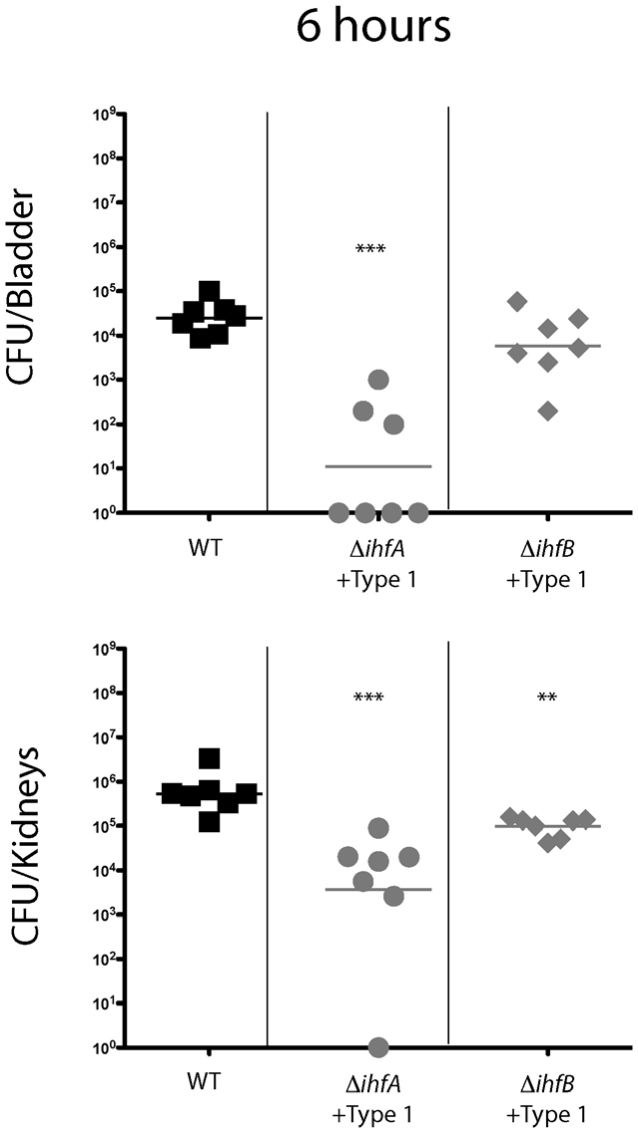
Type 1 piliation does not restore bladder colonization in the absence of IhfA. Each symbol represents single infected murine bladder or combined kidney pair. Female mice were infected with wild type UTI89/pMMB66 (black filled squares), ROL745/pMMB66 (UTI89 *ihfA11*; gray filled circles), or ROL603/pMMB66 (UTI89 *ΔihfB;* gray filled triangles) for 6 hours post inoculation. Bacterial burden of bladders and kidneys was enumerated as colony forming units ( CFUs). Statistical significance determined using non-parametric Mann Whitney (**<0.002, *** = 0.0006).

It is well established that the presence of the Type 1 pilus is an essential trait for both the initial adhesion to bladder epithelial cells and intracellular community [31]–[40]. The absence of piliation for strains defective in either Ihf subunit could account for the colonization defects observed. To bypass the requirement for IhfAB in the production of the Type 1 pilus and determine additional roles for the IhfAB subunits in during pathogenesis, an episomal copy of the *fim* operon under control of the P*_trc_* promoter was introduced into the mutant strains. The capacity to agglutinate yeast was fully restored in the mutant strains (Table 2). In the absence of IhfA, there was at least a 2 log decrease in bacterial burden, despite the presence of abundant Type 1 piliation (p<0.002)(Figure 6). There were no detectible bacteria in the majority of the bladders (4 of 7), suggesting that IhfA participates in additional processes during cystitis. There was approximately a 1 log decrease in the bacterial burden in the absence of IhfB that did not reach statistical significance (Figure 6). The recovery of virulence in the absence of IhfB suggests that the major defect is associated with loss of this subunit is the absence of Type 1 pili. These results suggest that there are unique functions for each of the Ihf subunits and that at least IhfA participates in functions in addition to piliation.

### IhfAB is present in extrabacterial milieu of IBCs

**Figure 7:**
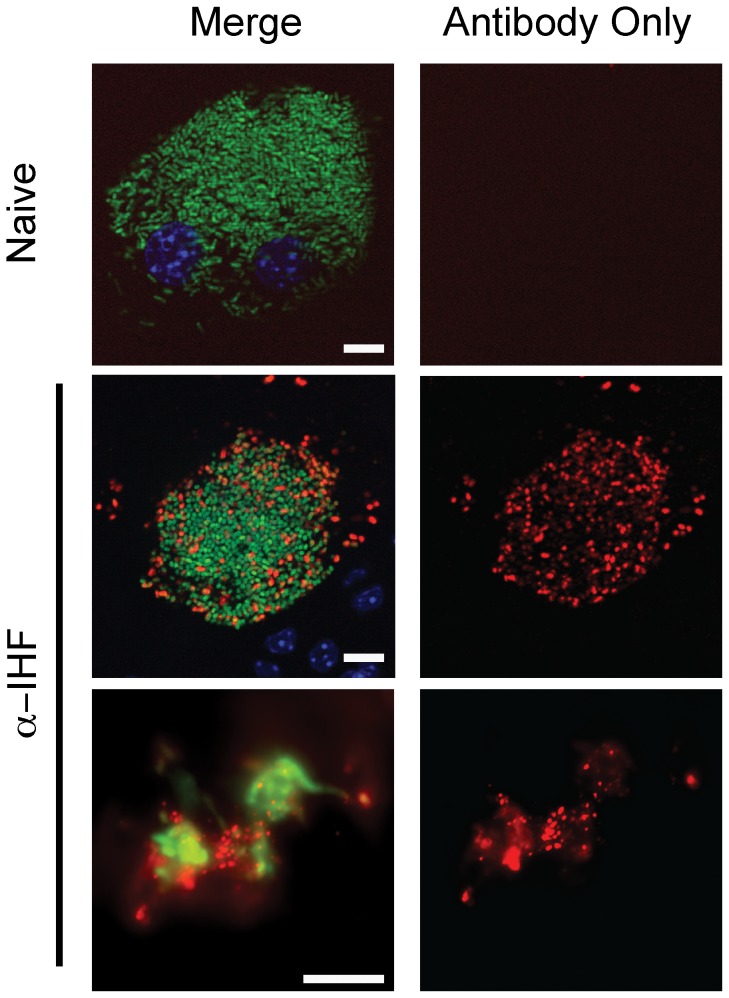
IhfAB is extrabacterial within the IBC milieu. Bladders were removed at 16 hours following transurethral introduction of UTI89/pANT4 (green). At 16 hours post introduction of bacteria, the bladders were harvested, bisected, fixed, host cells permeabilized and eIhfAB was observed with antibody (red) and Hoescht counterstained (blue). (A, D) No staining is observed when naïve rabbit serum is used as primary antibody. (B,E) eIhfAB is observed between the bacteria within the epithelial cell. (C, F) eIhfAB remains associated with UPEC following egress onto the surface of the bladder. Scale bar  = 10 µm.

We have previously demonstrated that addition of antibody directed against IhfAB will disrupt the integrity of communities of UPEC grown on glass surfaces [28], suggesting that eIhfAB is important for the stability of interbacterial interactions. In addition, little is known regarding the presence and nature of the IBC matrix. To determine whether IhfAB could be detected in the extrabacterial milieu of IBCs as an indicator for the presence of extrabacterial DNA, immunofluorescence microscopy was performed on infected, splayed whole mount mouse bladders. The epithelial cells were permeabilized under conditions that do not affect bacterial cell integrity (Methods and Materials). Evaluation of IBCs of UTI89 (green) at 16 hours post infection revealed the presence of eIhfAB (red) throughout the community architecture of established IBCs (Figure 7). In addition, eIhfAB is detected associated with UPEC that egress from infected epithelial cells (Figure 7). The punctate staining was not observed when naïve serum was used under the same experimental conditions. This evidence suggests that IhfAB is a component of the IBC matrix and that some of the phenotypes associated with the aberrant architecture may be associated with defects in the composition or deposition of eIhfAB in the IBC matrix.

### Antibody against DNABII family of proteins reduces UPEC binding to bladder epithelia

**Figure 8:**
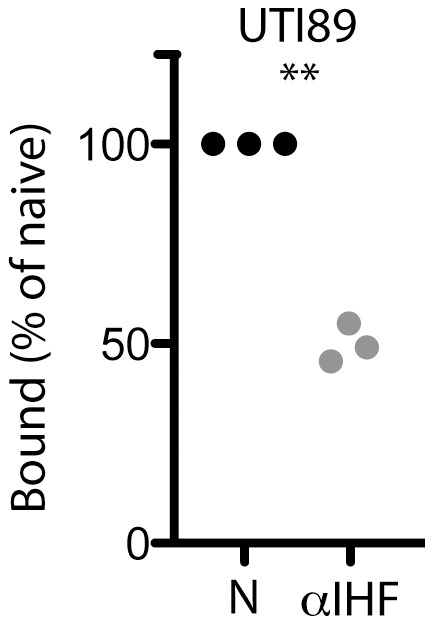
Antiserum against IhfAB reduces UPEC binding to cultured bladder epithelial cells. HTB-4 human bladder transitional carcinoma cell monolayers were infected with UTI89 treated with naïve antiserum (N; black) or specific antiserum directed against IhfAB (αIHF; gray). Each symbol represents the average of three experiments performed on the same day. Each experiment was replicated on three separate occasions. The number of bacteria bound is reported as a percentage of the bacteria bound in the presence of naïve serum. Statistical significance was determined by two-tailed Mann-Whitney test (**, p = 0.003).

To further validate the presence of eIhfAB associated with UPEC, we determined the ability of antisera to prevent binding of UPEC to bladder epithelial cells. We hypothesized that the steric hindrance imposed by antibody binding to eIhfAB would preclude UPEC type 1-mediated attachment to cultured human bladder epithelial cells. UTI89 was exposed to antisera directed against IhfAB prior to introduction to a monolayer of bladder epithelial cells. The presence of the antisera directed against IhfAB reduced the attachment of UTI89 (Figure 8) further supporting the observation of IhfAB on the bacterial surface.

### IhfA and IhfB are dispensable for early colonization of the kidney

In contrast to the bladder, where the IhfA and IhfB subunits are required for early stages of colonization, the absence of either the IhfA or IhfB subunit does not dramatically affect the colonization of the kidney at 6 hours post infection (Figure 2). Although statistical analysis demonstrated a significant difference, the biological relevance for less than a half-log difference in bacterial burden at this time point is unclear. The initial slight differences in colonization of the kidney may be indicative of the statistically significant two to four log decline in persistence observed with both mutant strains at 48 hours post infection (Figure 2; p<0.0006). As observed in the bladder tissue, addition of the gene in *trans* significantly increases the bacterial burden (Figure 2). The absence of either subunit was better tolerated in the kidney at 48 hours when these same mutants were essentially cleared from the bladder. IhfA alone supports more robust colonization of the kidney than does IhfB alone, suggesting that the subunits can at least function in the absence of the heterodimer in the kidney. The decreased persistence is likely not due to effects on motility as infection with strains defective in flagellum synthesis did not display a colonization defect under similar infection conditions [46] and Type 1 piliation was unable to restore the colonization of the kidney (p<0.002)(Figure 6). The P-pilus is known to bind to the globoseries glycoproteins on kidney epithelial cells [47]. Although the role of the P-pilus in adherence to the mouse kidney is unclear, the defect in colonization of the kidney and the predicted DNA bend in the promoter region of the *pap* operon [48] led us to evaluate the production of the P-pilus in our mutants. In contrast to the parental strain, neither mutant strain demonstrated hemagglutination following growth on tryptic soy agar (data not shown), suggesting that P-pilus (important for colonization of the kidney) also requires IhfAB.

### IhfAB protects the promoter region upstream of *papB*

**Figure 9:**
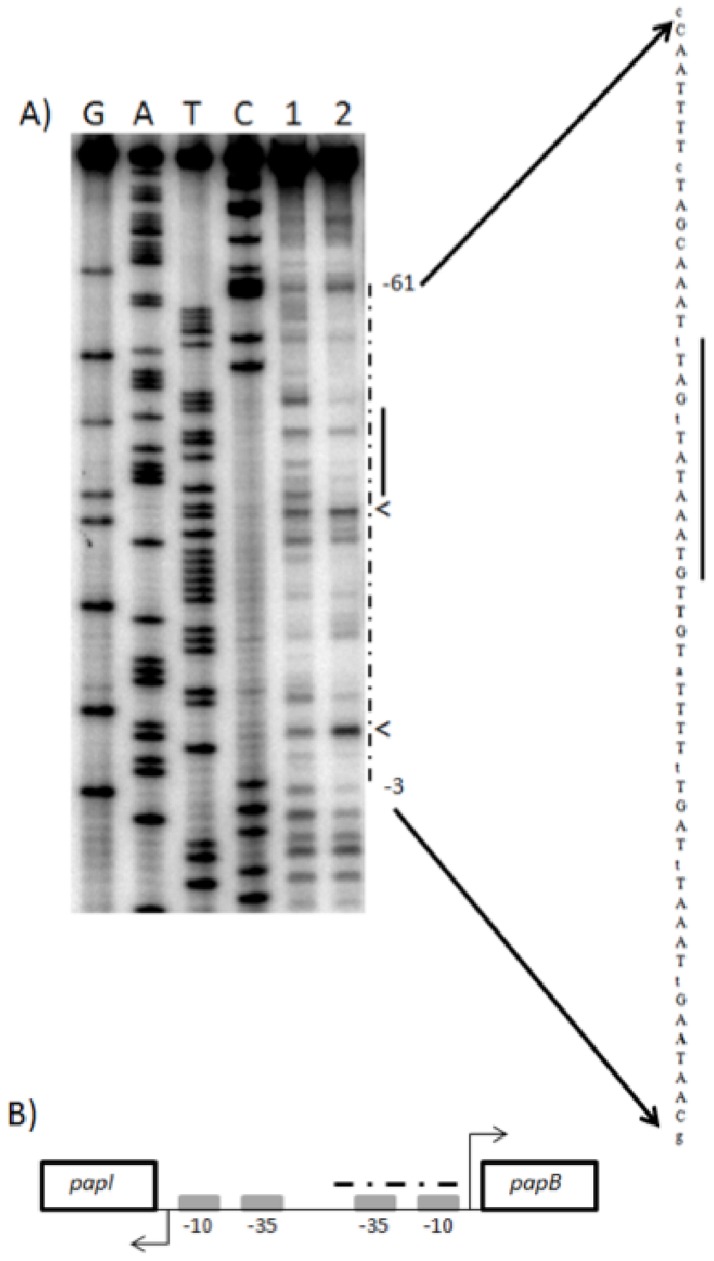
IHF binds the upstream region of *papB*. A) Lane 1–2: 0 & 50 nM IHF were incubated with labeled probe and subjected to DNase I digestion. The sequence protected by IHF is marked with the dashed line on the gel and the actual sequence is shown on the right side of the figure. The solid line on the sequence and gel represent the IHF recognition site, bold bases indicate the hypersensitive sites (< on the gel) and lower case letters are not protected. The numbers indicate the position from the transcription start (+1) of *papB*. B) Organization of the divergent *papB* and *papI* promoter region with the IHF protected region indicated by the dashed line.

As a first approach to determine whether IhfAB participates in regulation of the P-pilus, potential binding sites for IhfAB were evaluated in the promoter region of the operon encoding the genes for the P-pilus (*pap*). Using Regulatory Sequence Analysis DNA pattern matching Tools we found six sites with potential homology to the IhfAB consensus sequence (data not shown) in the 404 bp region between the divergent start sites of *papB* and *papI*. After closer examination of the critical residues for IHF-DNA association [49], we restricted our evaluation to two regions, 88 bp (AATCAATATTTAC) and 48 bp (TATCAACGGAAAG) from the transcriptional start of *papB* and *papI*, respectively. The site identified upstream of *papI* does not appear to serve as an IhfAB binding site based upon DNase I footprint analysis (data not shown). However, the sequence upstream of *papB,* was protected by the inclusion of IhfAB in the reaction, indicating that IhfAB binds to the promoter region of *papB* which encompasses the −35 region and −10 regions (Figure 9) [50], [51]. Immediately upstream of both the −35 and −10 region we observed sites of enhanced DNAseI digestion. The enhanced sensitivity to digestion is often the result of local DNA bending which allows greater access of DNaseI to the phosphodiester backbone and is indicative of induced structure of IhfAB [52].

## Discussion

Evaluation of multiple phenotypes associated with UPEC persistence during urinary tract infection demonstrates that the inactivation of *ihfA* and *ihfB* are not equivalent. Consistent with previous observations that homodimer species are active *in vitro* [29], [30], our data suggests that there may be independent functions for the homodimers under specific environmental conditions. For example, *ihfA* expression follows LexA-dependent activation by the SOS response [53]. We have demonstrated that the SOS response is activated in intracellular UPEC during bacterial cystitis [41], [45] and we would expect increased production of IhfA in the presence of host immune responses that include DNA damaging agents (i.e. reactive oxygen and nitrogen species) during infection. This increased production of IhfA could result in the presence of both IhfAB heterodimers and IhfA homodimers within the same bacterium during infection. The potential requirement for additional IhfA during the SOS response may partially explain the decreased persistence of UTI89 *ihfA11*::*Tn*10 during cystitis. IhfA is unstable under conditions of overproduction [54] and could contribute to the decreased persistence observed in the bladder. As we have demonstrated that individual subunits retain novel properties with regards to community development, it is possible that mixed homodimer and heterodimer populations may provide unique roles during the pathogenic lifestyle.

IhfAB is known to participate in the transcription of type 1 pili and capsule [21], [22]. Strains defective in production of the type 1 pilus demonstrate a reduction in the number of infected cells, and in the few cases where intracellular bacteria are detected, there is an absence of interbacterial interactions resulting in evenly distributed bacteria within the superficial bladder epithelial cell [32]. We demonstrated that replacement of Type 1 piliation did not restore virulence in the absence of IhfA. This is in contrast to recent reports where restoration of Type 1 piliation led to a recovery of virulence with other pleiotropic mutants [39], [40] when tested independently. In addition, we compared the intracellular phenotypes of the *ihfA* and *ihfB* mutants with strains deficient in type 1 pili and K1 capsule, where aberrant architecture of intracellular clusters was previously described [32], [33], [44]. UTI89 *ihfA11*::*Tn*10 retains interbacterial interactions during intracellular growth (Figure 3) and the more globular architecture of the prototypical IBC, but appear to have reduced growth and morphological changes as compared with the parent (Figure 5). Due to the few infected cells observed with the mutant strain, we were unable to determine the contribution of IhfA to the later developmental stages. However, the significant decrease in bacterial burden (Figure 2) suggests that the later stages of development are also compromised.

The phenotype of the UTI89 *ΔihfB* included the reduction in the number of intracellular clusters as well as a unique appearance in interbacterial interactions (Figure 3). The interbacterial spacing resembles that of UTI89 *surA* (defective in type 1 pili and outer membrane beta barrel proteins) [33], [55]–[57] but the overall shape of the cluster is different (Figure 5). The clusters formed by UTI89 *ΔihfB* are similar in overall shape with those of the UTI89 Δ*kpsF* strain (defective in the K1 capsule) (Figures 3, 5) [44], but lack the morphological changes associated with deficiencies in capsule production. Comparison of the architectural and growth characteristics of each UPEC mutant provides additional insight into the specific defects rather than just evaluation of bacterial burden alone.

The characterization of bacterial elements that are critical in the development of intracellular communities has been hampered by the severe bottleneck in the number of infectious organisms required to establish disease [58]. The use of microscopic evaluation of the intracellular clusters formed in the absence of SurA (another pleiotropic mutation)[33], led to the identification of Type 1 pilus and OmpA as critical complexes required for intracellular development [32], [59]. Along these lines, we herein describe a new binding site for IhfAB that contributes to regulation of the *pap* operon. Moreover, the observation that the defects in UPEC development are quantitatively different in the absence of either subunit using our newly developed software package [60] provides a platform for the identification of additional bacterial traits that are required for UPEC development.

The observation that IHF simultaneously participates in the virulence of UPEC by regulating transcription of key virulence traits intra-bacterially as well as contributing to the composition of the matrix extra-bacterially suggests that the use of therapeutics that target IHF function would severely cripple UPEC through inactivation of a number of traits that are critical for pathogenesis (i.e. type 1 pilus, P pilus, capsule, community integrity). Such a therapy could potentially affect both extrabacterial IhfAB and intrabacterial IhfAB targets of UPEC pathogenesis simultaneously and significantly reduce the probability for accumulation of resistant strains. Thus, further characterization of the bacterial traits influenced by IhfAB could lead to novel approaches to control UTIs.

## Materials and Methods

### Bacteria strains and plasmids

The strains and plasmids used in this study are indicated in Table 1. Constitutive synthesis of green fluorescent protein was performed using pANT4 (previously designated pCOMGFP) [2], [61]. Production of either or both IHF subunits was accomplished using pHNα [62], pHNβ (kindly provided by Howard Nash), or pHNαβ [63]. Ectopic production of the Type 1 pilus was accomplished using pMMB66 [64].

### Media and growth

Bacteria were grown at 37°C in Luria-Bertani (LB; Fisher Scientific, Pittsburg, PA) broth in the absence of aeration unless otherwise indicated (planktonic). For assessment of community development *in vitro*, *E. coli* strains indicated were prepared as described [28]. When appropriate, antibiotics were used at the following concentrations: 30 µg/ml kanamycin, 25 µg/ml chloramphenicol, 25 µg/ml tetracycline hydrochloride, or 100 µg/ml ampicillin (Fisher Scientific, Pittsburg, PA).

### The *fimS* orientation

Twenty-five microliters of overnight liquid cultures as well as *in vitro* community cultures [28] of various growth phases of planktonic and biofilms were lysed, and the supernatants containing chromosomal DNA were used for amplification of the *fimS* region. The amplification products were digested with BstUI (New England Biolabs) and visualized following separation on a 2% agarose gel as described previously [65] to determine the orientation (“off”  = 539 & 187 bp, while “on”  = 433 & 293 bp) of the promoter.

### Inactivation of *ihfA* and *ihfB* in MG1655 & UTI89

Laboratory strains of *E. coli* that carry the individual mutations were obtained from various sources (Table 1). The mutations were introduced into the prototypical UPEC strain, UTI89, and a laboratory adapted strain, MG1655, from the laboratory strains by P1 transduction [66]. To facilitate P1 binding to UPEC, cultures were grown at room temperature to reduce production of the K1 capsule [67]. In all cases, at least three independent transductants were tested in all laboratory-based assays.

### *in vitro* yeast agglutination assay

The mannose-sensitive Yeast Agglutination (YA) assay (type 1 piliation) or the hemagglutination assay (P piliation) was performed as previously described [45], [68].

### Mouse infections

UTI89, ROL745 (UTI89 *ihfA11::Tn*10) and ROL603 (UTI89 Δ*ihfB*) were grown in LB broth in the absence of aeration for 16 hours to an OD_600_ ∼1.2 at 37°C. Inoculum of each strain was normalized such that ∼10^7^ bacteria were transurethrally introduced directly to the bladders of female C3H/HeNHsd mice (Figure 2: Harlan Sprague Laboratory; Indianapolis, IN) (Figure 6: Charles River Laboratory, Wilmington, MA) as previously described [2], [3], [69]. Tissues were harvested at times indicated post-inoculation. Enumeration of bacterial burden was performed as previously described [33], [41]. Statistical significance was determined using a two-tailed Mann Whitney test of the wild type UTI89 for the comparison with each UPEC mutant individually. The experiments were performed in cohorts of 5 mice and repeated on separate occasions. All data points are presented. Maintenance of all mice was in strict accordance of the Institutional Animal Care and Use Committee (IACUC) rules and regulations. All animals are housed in accordance with USDA guidelines for the care and housing of laboratory animals, and USDA officials routinely inspect the facilities. The experiments presented in this manuscript are approved (AR06-00119) by The Research Institute at Nationwide Children's Hospital Institutional Laboratory Animal Care and Use Committee (Welfare Assurance Number A3544-01).

### Immunofluorescence microscopy of whole mount tissue

Visualization of UPEC colonization was performed as previously described [33], [41]. Bladders were harvested and stretched followed by 4% PFA with 0.1% Triton X-100 in PBS overnight to permeabilize the epithelial plasma membranes. Bacterial and host DNA were visualized by the addition of Hoechst 34580 (Invitrogen; Carlsbad, CA) for 10 minutes. Bladders were stained as indicated below and mounted with ProLong Gold antifade reagent (Invitrogen, Carlsbad, CA). Images were acquired using an Axiovert 200 M inverted epifluorescence microscope equipped with a motorized stage, an Axiocam MRM CCD camera and the Apotome component to improve fluorescence resolution (Carl Zeiss, INC, Thornwood, NY). The levels of the fluorescent images were adjusted to all pixels within the image using Adobe Photoshop (Adobe Systems Incorporated; San Jose, CA). Quantitation of bacterial morphologies was performed using Image J (developed at the National Institutes of Health, http://rsbweb.nih.gov/ij/) and ProkaryMetrics [60].

### Visualization of UPEC

Intracellular UPEC were visualized the addition of Rabbit Anti-*E.coli* polyclonal antisera (PAb) (USbiological, MA) diluted 1∶200 in PBS with 0.1% Triton X-100 at 37°C for 1 hour. Splayed bladders were washed by PBS for 3 times before the addition of Goat anti Rabbit secondary IgG conjucated to Alexa-594 (Life Technologies) 1∶200 with 0.1% Triton X-100 at 37°C for 1 hour. Residual antibodies were removed by washing with PBS for 3 times before stained by Hoechst 34580 (Life Technologies, Carlsbad, CA).

### Visualization of extrabacterial UPEC in IBCs

Antisera raised against purified recombinant IHF [62] or naïve rabbit serum was diluted 1∶100 in PBS and applied to mounted bladder tissues for 1 hour. The presence of specific antibody was visualized using Alexa 594-conjugated anti-IgG antibodies (Life Technologies, Carlsbad, CA).

### Binding to bladder epithelium in the presence or absence of antisera directed against IhfAB

Overnight cultures of UTI89 was diluted to ∼1×10^5^ colony forming units ( CFU)/ml in minimal essential medium (ATCC; Manassas, VA). A 1∶50 dilution of antiserum raised against IhfAB [62] was added to the culture for 5 minutes. The medium was then overlayed onto a confluent monolayer of HTB-4 human bladder transitional carcinoma cells (ATCC; Manassas, VA). Binding was facilitated by centrifugation before cells were washed, lysed and bound then the bacteria were enumerated as previously described [33].

### Identification of IHF recognition sites

Using available Regulatory Sequence Analysis DNA pattern matching tools (RSAT, http://rsat.ulb.ac.be/rsat/) we analyzed the divergent *papB* and *papI* promoter region for the presence of IHF recognition sites (WATCAANNNNTTR, where W = A or T, R = G or A, N =  any nucleotide) [49].

### DNaseI Footprinting Assay

To label the DNA substrates 1 µM oSG759 (5′ ATGTTTCCCCCTTCTGTCG 3′) was labeled at the 5′-end with 0.85 µM of [γ-32P] ATP (10 mCi/ml, New Life Science Products) using T4 polynucleotide kinase (Promega). To amplify the promoter region of *papB* (141 bp) labeled oSG759 was used along with the unlabeled primer oSG760 (5′ TCTGGTTTGGTTTTGTTTTGC 3′) in a PCR reaction using purified *E. coli* UTI89 chromosomal DNA. Identification of residues protected by IHF binding was performed as previously described [70]. Briefly, the labeled DNA substrate was incubated with 50 nM purified recombinant IhfAB [54] for 30 min at room temperature in footprinting buffer (25 mM Tris-HCl, pH 7.5, 50 mM KCl, 6.25 mM MgCl_2_, 10% glycerol, 1 mM DTT) before the addition of 2.5 mM CaCl_2_ and 5 mM MgCl_2_. Partial digestion using DNaseI (Promega, Madison, Wisconsin) was allowed for 1 min. DNA was extracted with phenol:chloroform, ethanol-precipitated and resuspended in Sequitherm EXCEL II DNA sequencing stop buffer (Epicentre Biotechnologies). A sequence ladder for the *papB* fragments was generated using labeled oSG759, the *papB* promoter fragment and the Sequitherm EXCEL II DNA sequencing kit (Epicentre Biotechnologies) following the manufacturer's instructions. All reactions were separated on a 6% sequencing gel run at 40 V/cm for 1.5 hours. The gel was then dried and scanned with the Typhoon imaging system (GE Healthcare).

## References

[1] Foxman B (2010) The epidemiology of urinary tract infection. Nature reviews Urology 7: 653–660. 21139641 10.1038/nrurol.2010.190

[2] Justice SS, Hung C, Theriot JA, Fletcher DA, Anderson GG, et al (2004) From the cover: Differentiation and developmental pathways of uropathogenic *Escherichia coli* in urinary tract pathogenesis. Proceedings of the National Academy of Sciences of the United States of America 101: 1333–1338. 14739341 10.1073/pnas.0308125100 PMC337053

[3] Mulvey MA, Lopez-Boado YS, Wilson CL, Roth R, Parks WC, et al (1998) Induction and evasion of host defenses by type 1-piliated uropathogenic *Escherichia coli* . Science 282: 1494–1497. 9822381 10.1126/science.282.5393.1494

[4] Anderson GG, Palermo JJ, Schilling JD, Roth R, Heuser J, et al (2003) Intracellular bacterial biofilm-like pods in urinary tract infections. Science 301: 105–107. 12843396 10.1126/science.1084550

[5] Bishop BL, Duncan MJ, Song J, Li G, Zaas D, et al (2007) Cyclic AMP-regulated exocytosis of *Escherichia coli* from infected bladder epithelial cells. Nat Med 13: 625–630. 17417648 10.1038/nm1572

[6] Song J, Bishop BL, Li G, Duncan MJ, Abraham SN (2007) TLR4 initiated and cAMP mediated abrogation of bacterial invasion of the bladder. Cell Host Microbe 1: 287–298. 17710226 10.1016/j.chom.2007.05.007 PMC1950120

[7] Eto DS, Jones TA, Sundsbak JL, Mulvey MA (2007) Integrin-Mediated Host Cell Invasion by Type 1-Piliated Uropathogenic *Escherichia coli* . PLoS Pathog 3: e100. 17630833 10.1371/journal.ppat.0030100 PMC1914067

[8] Mulvey MA, Schilling JD, Hultgren SJ (2001) Establishment of a persistent *Escherichia coli* reservoir during the acute phase of a bladder infection. Infect Immun 69: 4572–4579. 11402001 10.1128/IAI.69.7.4572-4579.2001 PMC98534

[9] Mysorekar IU, Hultgren SJ (2006) Mechanisms of uropathogenic *Escherichia coli* persistence and eradication from the urinary tract. Proc Natl Acad Sci U S A 103: 14170–14175. 16968784 10.1073/pnas.0602136103 PMC1564066

[10] Hannan TJ, Mysorekar IU, Hung CS, Isaacson-Schmid ML, Hultgren SJ (2010) Early severe inflammatory responses to uropathogenic *E. coli* predispose to chronic and recurrent urinary tract infection. PLoS Pathog 6. 10.1371/journal.ppat.1001042 PMC2930321 20811584

[11] Rosen DA, Hooton TM, Stamm WE, Humphrey PA, Hultgren SJ (2007) Detection of intracellular bacterial communities in human urinary tract infection. PLoS Med 4: e329. 18092884 10.1371/journal.pmed.0040329 PMC2140087

[12] Rosen DA, Pinkner JS, Jones JM, Walker JN, Clegg S, et al (2008) Utilization of an intracellular bacterial community pathway in *Klebsiella pneumoniae* urinary tract infection and the effects of FimK on type 1 pilus expression. Infect Immun 76: 3337–3345. 18411285 10.1128/IAI.00090-08 PMC2446714

[13] Rouviere-Yaniv J, Gros F (1975) Characterization of a novel, low-molecular-weight DNA-binding protein from *Escherichia coli* . Proceedings of the National Academy of Sciences of the United States of America 72: 3428–3432. 1103148 10.1073/pnas.72.9.3428 PMC433007

[14] Miller HI, Kikuchi A, Nash HA, Weisberg RA, Friedman DI (1979) Site-specific recombination of bacteriophage lambda: the role of host gene products. Cold Spring Harbor symposia on quantitative biology 43 Pt 2: 1121–1126. 10.1101/sqb.1979.043.01.125 158465

[15] Swinger KK, Rice PA (2004) IHF and HU: flexible architects of bent DNA. Current opinion in structural biology 14: 28–35. 15102446 10.1016/j.sbi.2003.12.003

[16] Jeong HS, Kim SM, Lim MS, Kim KS, Choi SH (2010) Direct interaction between quorum-sensing regulator SmcR and RNA polymerase is mediated by integration host factor to activate vvpE encoding elastase in *Vibrio vulnificus* . The Journal of biological chemistry 285: 9357–9366. 20110369 10.1074/jbc.M109.089987 PMC2843184

[17] Stonehouse E, Kovacikova G, Taylor RK, Skorupski K (2008) Integration host factor positively regulates virulence gene expression in *Vibrio cholerae* . Journal of bacteriology 190: 4736–4748. 18456804 10.1128/JB.00089-08 PMC2446820

[18] Li M, Rosenshine I, Tung SL, Wang XH, Friedberg D, et al (2004) Comparative proteomic analysis of extracellular proteins of enterohemorrhagic and enteropathogenic *Escherichia coli* strains and their *ihf* and *ler* mutants. Applied and environmental microbiology 70: 5274–5282. 15345410 10.1128/AEM.70.9.5274-5282.2004 PMC520853

[19] Morash MG, Brassinga AK, Warthan M, Gourabathini P, Garduno RA, et al (2009) Reciprocal expression of integration host factor and HU in the developmental cycle and infectivity of *Legionella pneumophila* . Applied and environmental microbiology 75: 1826–1837. 19201975 10.1128/AEM.02756-08 PMC2663186

[20] Hill SA, Samuels DS, Nielsen C, Knight SW, Pagotto F, et al (2002) Integration host factor interactions with *Neisseria* gene sequences: correlation between predicted binding sites and *in vitro* binding of Neisseria -derived IHF protein. Molecular and cellular probes 16: 153–158. 12030765 10.1006/mcpr.2001.0403

[21] Rowe S, Hodson N, Griffiths G, Roberts IS (2000) Regulation of the *Escherichia coli* K5 capsule gene cluster: evidence for the roles of H-NS, BipA, and integration host factor in regulation of group 2 capsule gene clusters in pathogenic *E. coli* . Journal of bacteriology 182: 2741–2745. 10781541 10.1128/jb.182.10.2741-2745.2000 PMC101981

[22] Corcoran CP, Dorman CJ (2009) DNA relaxation-dependent phase biasing of the fim genetic switch in *Escherichia coli* depends on the interplay of H-NS, IHF and LRP. Molecular microbiology 74: 1071–1082. 19889099 10.1111/j.1365-2958.2009.06919.x

[23] Winters BD, Ramasubbu N, Stinson MW (1993) Isolation and characterization of a *Streptococcus pyogenes* protein that binds to basal laminae of human cardiac muscle. Infection and immunity 61: 3259–3264. 8335359 10.1128/iai.61.8.3259-3264.1993 PMC280997

[24] Lunsford RD, Nguyen N, London J (1996) DNA-binding activities in *Streptococcus gordonii*: identification of a receptor-nickase and a histonelike protein. Current microbiology 32: 95–100. 8574134 10.1007/s002849900017

[25] Kim N, Weeks DL, Shin JM, Scott DR, Young MK, et al (2002) Proteins released by *Helicobacter pylori in vitro* . Journal of bacteriology 184: 6155–6162. 12399485 10.1128/JB.184.22.6155-6162.2002 PMC151949

[26] Zhang L, Ignatowski TA, Spengler RN, Noble B, Stinson MW (1999) Streptococcal histone induces murine macrophages To produce interleukin-1 and tumor necrosis factor alpha. Infection and immunity 67: 6473–6477. 10569765 10.1128/iai.67.12.6473-6477.1999 PMC97057

[27] Flemming HC, Wingender J (2010) The biofilm matrix. Nature reviews Microbiology 8: 623–633. 20676145 10.1038/nrmicro2415

[28] Goodman SD, Obergfell KP, Jurcisek JA, Novotny LA, Downey JS, et al (2011) Biofilms can be dispersed by focusing the immune system on a common family of bacterial nucleoid-associated proteins. Mucosal immunology 4: 625–637. 21716265 10.1038/mi.2011.27

[29] Werner MH, Clore GM, Gronenborn AM, Nash HA (1994) Symmetry and asymmetry in the function of *Escherichia coli* integration host factor: implications for target identification by DNA-binding proteins. Current biology : CB 4: 477–487. 7922368 10.1016/s0960-9822(00)00108-1

[30] Zulianello L, de la Gorgue de Rosny E, van Ulsen P, van de Putte P, Goosen N (1994) The HimA and HimD subunits of integration host factor can specifically bind to DNA as homodimers. The EMBO journal 13: 1534–1540. 8156991 10.1002/j.1460-2075.1994.tb06415.x PMC394982

[31] Hultgren SJ, Porter TN, Schaeffer AJ, Duncan JL (1985) Role of type 1 pili and effects of phase variation on lower urinary tract infections produced by *Escherichia coli.* . Infection and immunity 50: 370–377. 2865209 10.1128/iai.50.2.370-377.1985 PMC261959

[32] Wright KJ, Seed PC, Hultgren SJ (2007) Development of intracellular bacterial communities of uropathogenic *Escherichia coli* depends on type 1 pili. Cell Microbiol 9: 2230–2241. 17490405 10.1111/j.1462-5822.2007.00952.x

[33] Justice SS, Lauer SR, Hultgren SJ, Hunstad DA (2006) Maturation of intracellular *Escherichia coli* communities requires SurA. Infect Immun 74: 4793–4800. 16861667 10.1128/IAI.00355-06 PMC1539609

[34] Snyder JA, Lloyd AL, Lockatell CV, Johnson DE, Mobley HL (2006) Role of phase variation of type 1 fimbriae in a uropathogenic *Escherichia coli* cystitis isolate during urinary tract infection. Infect Immun 74: 1387–1393. 16428790 10.1128/IAI.74.2.1387-1393.2006 PMC1360342

[35] Bahrani-Mougeot FK, Buckles EL, Lockatell CV, Hebel JR, Johnson DE, et al (2002) Type 1 fimbriae and extracellular polysaccharides are preeminent uropathogenic *Escherichia coli* virulence determinants in the murine urinary tract. Mol Microbiol 45: 1079–1093. 12180926 10.1046/j.1365-2958.2002.03078.x

[36] Martinez JJ, Mulvey MA, Schilling JD, Pinkner JS, Hultgren SJ (2000) Type 1 pilus-mediated bacterial invasion of bladder epithelial cells. Embo J 19: 2803–2812. 10856226 10.1093/emboj/19.12.2803 PMC203355

[37] Chen SL, Hung CS, Pinkner JS, Walker JN, Cusumano CK, et al (2009) Positive selection identifies an *in vivo* role for FimH during urinary tract infection in addition to mannose binding. Proc Natl Acad Sci U S A 106: 22439–22444. 20018753 10.1073/pnas.0902179106 PMC2794649

[38] Cusumano CK, Pinkner JS, Han Z, Greene SE, Ford BA, et al (2011) Treatment and prevention of urinary tract infection with orally active FimH inhibitors. Sci Transl Med 3: 109ra115. 10.1126/scitranslmed.3003021 PMC3694776 22089451

[39] Crepin S, Houle S, Charbonneau ME, Mourez M, Harel J, et al (2012) Decreased Expression of Type 1 Fimbriae by a pst Mutant of Uropathogenic *Escherichia coli* Reduces Urinary Tract Infection. Infect Immun 80: 2802–2815. 22665376 10.1128/IAI.00162-12 PMC3434566

[40] Kostakioti M, Hadjifrangiskou M, Cusumano CK, Hannan TJ, Janetka JW, et al (2012) Distinguishing the Contribution of Type 1 Pili from That of Other QseB-Misregulated Factors when QseC Is Absent during Urinary Tract Infection. Infect Immun 80: 2826–2834. 22665375 10.1128/IAI.00283-12 PMC3434567

[41] Justice SS, Hunstad DA, Seed PC, Hultgren SJ (2006) Filamentation by *Escherichia coli* subverts innate defenses during urinary tract infection. Proc Natl Acad Sci U S A 103: 19884–19889. 17172451 10.1073/pnas.0606329104 PMC1750882

[42] Horvath DJ Jr, Li B, Casper T, Partida-Sanchez S, Hunstad DA, et al (2011) Morphological plasticity promotes resistance to phagocyte killing of uropathogenic *Escherichia coli* . Microbes Infect 13: 426–437. 21182979 10.1016/j.micinf.2010.12.004 PMC3071881

[43] Hunstad DA, Justice SS (2010) Intracellular lifestyles and immune evasion strategies of uropathogenic Escherichia coli. Annu Rev Microbiol 64: 203–221. 20825346 10.1146/annurev.micro.112408.134258

[44] Anderson GG, Goller CC, Justice S, Hultgren SJ, Seed PC (2011) Polysaccharide Capsule and Sialic Acid-Mediated Regulation Promote Biofilm-like Intracellular Bacterial Communities During Cystitis. Infect Immun 78: 963–975. 10.1128/IAI.00925-09 PMC2825929 20086090

[45] Li B, Smith P, Horvath DJ Jr, Romesberg F, Justice SS (2010) SOS regulatory elements are essential for UPEC pathogenesis. Microbes Infect 12: 662–668. 20435157 10.1016/j.micinf.2010.04.009

[46] Wright KJ, Seed PC, Hultgren SJ (2005) Uropathogenic *Escherichia coli* flagella aid in efficient urinary tract colonization. Infect Immun 73: 7657–7668. 16239570 10.1128/IAI.73.11.7657-7668.2005 PMC1273872

[47] Stromberg N, Marklund BI, Lund B, Ilver D, Hamers A, et al (1990) Host-specificity of uropathogenic *Escherichia coli* depends on differences in binding specificity to Gal alpha 1-4Gal-containing isoreceptors. EMBO J 9: 2001–2010. 1693334 10.1002/j.1460-2075.1990.tb08328.x PMC551909

[48] Hung DL, Raivio TL, Jones CH, Silhavy TJ, Hultgren SJ (2001) Cpx signaling pathway monitors biogenesis and affects assembly and expression of P pili. Embo J 20: 1508–1518. 11285215 10.1093/emboj/20.7.1508 PMC145513

[49] Hales LM, Gumport RI, Gardner JF (1994) Determining the DNA sequence elements required for binding integration host factor to two different target sites. Journal of bacteriology 176: 2999–3006. 8188600 10.1128/jb.176.10.2999-3006.1994 PMC205457

[50] Baga M, Goransson M, Normark S, Uhlin BE (1985) Transcriptional activation of a pap pilus virulence operon from uropathogenic *Escherichia coli* . The EMBO journal 4: 3887–3893. 2868893 10.1002/j.1460-2075.1985.tb04162.x PMC554745

[51] Goransson M, Forsman P, Nilsson P, Uhlin BE (1989) Upstream activating sequences that are shared by two divergently transcribed operons mediate cAMP-CRP regulation of pilus-adhesin in *Escherichia coli* . Molecular microbiology 3: 1557–1565. 2575704 10.1111/j.1365-2958.1989.tb00141.x

[52] Suck D (1997) DNA recognition by structure-selective nucleases. Biopolymers 44: 405–421. 9782777 10.1002/(SICI)1097-0282(1997)44:4<405::AID-BIP5>3.0.CO;2-L

[53] Miller HI, Kirk M, Echols H (1981) SOS induction and autoregulation of the himA gene for site-specific recombination in *Escherichia coli* . Proceedings of the National Academy of Sciences of the United States of America 78: 6754–6758. 6796964 10.1073/pnas.78.11.6754 PMC349128

[54] Nash HA, Robertson CA, Flamm E, Weisberg RA, Miller HI (1987) Overproduction of *Escherichia coli* integration host factor, a protein with nonidentical subunits. Journal of bacteriology 169: 4124–4127. 3305480 10.1128/jb.169.9.4124-4127.1987 PMC213718

[55] Justice SS, Hunstad DA, Harper JR, Duguay AR, Pinkner JS, et al (2005) Periplasmic peptidyl prolyl *cis-trans* isomerases are not essential for viability, but SurA is required for pilus biogenesis in *Escherichia coli* . J Bacteriol 187: 7680–7686. 16267292 10.1128/JB.187.22.7680-7686.2005 PMC1280321

[56] Rouviere PE, Gross CA (1996) SurA, a periplasmic protein with peptidyl-prolyl isomerase activity, participates in the assembly of outer membrane porins. Genes & development 10: 3170–3182. 8985185 10.1101/gad.10.24.3170

[57] Lazar SW, Kolter R (1996) SurA assists the folding of *Escherichia coli* outer membrane proteins. Journal of bacteriology 178: 1770–1773. 8626309 10.1128/jb.178.6.1770-1773.1996 PMC177866

[58] Schwartz DJ, Chen SL, Hultgren SJ, Seed PC (2011) Population dynamics and niche distribution of uropathogenic *Escherichia coli* during acute and chronic urinary tract infection. Infection and immunity 79: 4250–4259. 21807904 10.1128/IAI.05339-11 PMC3187256

[59] Nicholson TF, Watts KM, Hunstad DA (2009) OmpA of uropathogenic *Escherichia coli* promotes postinvasion pathogenesis of cystitis. Infect Immun 77: 5245–5251. 19797074 10.1128/IAI.00670-09 PMC2786482

[60] Dabdoub S, VanderBrink BA, Justice SS, Ray WC (2012) Quantitating pathogenic biofilm architecture in biopsied tissue. Visual Analytics in Healthcare in press.

[61] Lee AK, Falkow S (1998) Constitutive and inducible green fluorescent protein expression in *Bartonella henselae* . Infection and immunity 66: 3964–3967. 9673287 10.1128/iai.66.8.3964-3967.1998 PMC108464

[62] Granston AE, Nash HA (1993) Characterization of a set of integration host factor mutants deficient for DNA binding. Journal of molecular biology 234: 45–59. 8230206 10.1006/jmbi.1993.1562

[63] Lee EC, Hales LM, Gumport RI, Gardner JF (1992) The isolation and characterization of mutants of the integration host factor (IHF) of *Escherichia coli* with altered, expanded DNA-binding specificities. The EMBO journal 11: 305–313. 1531459 10.1002/j.1460-2075.1992.tb05053.x PMC556451

[64] Barnhart MM, Sauer FG, Pinkner JS, Hultgren SJ (2003) Chaperone-subunit-usher interactions required for donor strand exchange during bacterial pilus assembly. J Bacteriol 185: 2723–2730. 12700251 10.1128/JB.185.9.2723-2730.2003 PMC154394

[65] Smith SG, Dorman CJ (1999) Functional analysis of the FimE integrase of *Escherichia coli* K-12: isolation of mutant derivatives with altered DNA inversion preferences. Molecular microbiology 34: 965–979. 10594822 10.1046/j.1365-2958.1999.01657.x

[66] Silhavy TJ, Berman ML, Enquist LW (1984) Experiments with Gene Fusions. Cold Spring Harbor, NY: Cold Spring Harbor Laboratory.111–113 p.

[67] Bortolussi R, Ferrieri P, Quie PG (1983) Influence of growth temperature of *Escherichia coli* on K1 capsular antigen production and resistance to opsonization. Infection and immunity 39: 1136–1141. 6341228 10.1128/iai.39.3.1136-1141.1983 PMC348074

[68] Hultgren SJ, Duncan JL, Schaeffer AJ, Amundsen SK (1990) Mannose-sensitive haemagglutination in the absence of piliation in *Escherichia coli* . Molecular microbiology 4: 1311–1318. 1980711 10.1111/j.1365-2958.1990.tb00710.x

[69] Hung CS, Dodson KW, Hultgren SJ (2009) A murine model of urinary tract infection. Nat Protoc 4: 1230–1243. 19644462 10.1038/nprot.2009.116 PMC2963178

[70] Hung DC, Downey JS, Ayala EA, Kreth J, Mair R, et al (2011) Characterization of DNA binding sites of the ComE response regulator from *Streptococcus mutans* . Journal of bacteriology 193: 3642–3652. 21602345 10.1128/JB.00155-11 PMC3133340

[71] Baba T, Ara T, Hasegawa M, Takai Y, Okumura Y, et al.. (2006) Construction of Escherichia coli K-12 in-frame, single-gene knockout mutants: the Keio collection. Molecular systems biology 2: 2006 0008. 10.1038/msb4100050 PMC1681482 16738554

[72] Blattner FR, Plunkett G 3rd, Bloch CA, Perna NT, Burland V, et al (1997) The complete genome sequence of *Escherichia coli* K-12. Science 277: 1453–1462. 9278503 10.1126/science.277.5331.1453

[73] Mizusawa H, Lee CH, Kakefuda T (1981) Alteration of plasmid DNA-mediated transformation and mutation induced by covalent binding of benzo[alpha]pyrene-7,8-dihydrodiol-9,10-oxide in *Escherichia coli* . Mutation research 82: 47–57. 6267457 10.1016/0027-5107(81)90137-8

[74] Flamm EL, Weisberg RA (1985) Primary structure of the hip gene of *Escherichia coli* and of its product, the beta subunit of integration host factor. Journal of molecular biology 183: 117–128. 3159903 10.1016/0022-2836(85)90206-2

[75] Miller HI (1984) Primary structure of the himA gene of *Escherichia coli*: homology with DNA-binding protein HU and association with the phenylalanyl-tRNA synthetase operon. Cold Spring Harbor symposia on quantitative biology 49: 691–698. 6397321 10.1101/sqb.1984.049.01.078

[76] Hunstad DA, Justice SS, Hung CS, Lauer SR, Hultgren SJ (2005) Suppression of bladder epithelial cytokine responses by uropathogenic *Escherichia coli* . Infect Immun 73: 3999–4006. 15972487 10.1128/IAI.73.7.3999-4006.2005 PMC1168571

